# Ultrasonographic features of adrenal gland lesions in dogs can aid in diagnosis

**DOI:** 10.1186/s12917-016-0895-1

**Published:** 2016-11-28

**Authors:** Elena Pagani, Massimiliano Tursi, Chiara Lorenzi, Alberto Tarducci, Barbara Bruno, Enrico Corrado Borgogno Mondino, Renato Zanatta

**Affiliations:** 1 Department of Veterinary Sciences, University of Turin, Largo Paolo Braccini 2-5, 10095 Grugliasco, TO Italy; 2 Department of Agriculture, Forest and Food Sciences, University of Turin, L. Paolo Braccini, 2, 10095 Grugliasco, TO Italy

**Keywords:** Adrenal gland, Ultrasonography, Dogs, Lesion, Tumor

## Abstract

**Background:**

Ultrasonography to visualize adrenal gland lesions and evaluate incidentally discovered adrenal masses in dogs has become more reliable with advances in imaging techniques. However, correlations between sonographic and histopathological changes have been elusive. The goal of our study was to investigate which ultrasound features of adrenal gland abnormalities could aid in discriminating between benign and malignant lesions. To this end, we compared diagnosis based on ultrasound appearance and histological findings and evaluated ultrasound criteria for predicting malignancy.

**Results:**

Clinical records of 119 dogs that had undergone ultrasound adrenal gland and histological examination were reviewed. Of these, 50 dogs had normal adrenal glands whereas 69 showed pathological ones. Lesions based on histology were classified as cortical adrenal hyperplasia (*n* = 67), adenocarcinoma (*n* = 17), pheochromocytoma (*n* = 10), metastases (*n* = 7), adrenal adenoma (*n* = 4), and adrenalitis (*n* = 4). Ultrasonographic examination showed high specificity (100%) but low sensitivity (63.7%) for identifying the adrenal lesions, which improved with increasing lesion size. Analysis of ultrasonographic predictive parameters showed a significant association between lesion size and malignant tumors. All adrenal gland lesions >20 mm in diameter were histologically confirmed as malignant neoplasms (pheochromocytoma and adenocarcinoma). Vascular invasion was a specific but not sensitive predictor of malignancy. As nodular shape was associated with benign lesions and irregular enlargement with malignant ones, this parameter could be used as diagnostic tool. Bilaterality of adrenal lesions was a useful ultrasonographic criterion for predicting benign lesions, as cortical hyperplasia.

**Conclusions:**

Abnormal appearance of structural features on ultrasound images (e.g., adrenal gland lesion size, shape, laterality, and echotexture) may aid in diagnosis, but these features alone were not pathognomic. Lesion size was the most direct ultrasound predictive criterion. Large and irregular masses seemed to be better predictors of malignant neoplasia and lesions <20 mm in diameter and nodular in shape were often identified as cortical hyperplastic nodules or adenomas.

## Background

In human medicine, the use of advanced imaging techniques (e.g., ultrasound, computed tomography, scintigraphy, and magnetic resonance imaging) has resulted in the increasing identification of incidental adrenal gland lesions [1]. Among the methods of diagnostic imaging, ultrasound is considered a relatively rapid, non-invasive, inexpensive and reliable modality to evaluate suspected adrenal masses [2]. Beginning in the mid-1990s, ultrasound imaging has become a widely used diagnostic tool in veterinary medicine. Its application in small animal practice has yielded extensive information on normal adrenal gland appearance in the dog [3–10], but it has also led to an increase in the incidental discovery of adrenal masses. Though a common occurrence in diagnostic imaging, adrenal masses can constitute a significant clinical dilemma in the dog [11]. They may be benign (e.g., hyperplasia, myelolipoma, cortical adenoma) or malignant (e.g., pheochromocytoma, cortical carcinoma, and metastases) [12]. Adrenal gland lesions (nodules and masses) commonly develop in older dogs, but surgery is indicated in only a small fraction of such cases, usually in malignant and hormone-secreting tumors [13]. While ultrasonography is an effective method for localizing adrenal lesions, standardized ultrasound criteria to distinguish benign from malignant lesions or functional cortisol-secreting from non-functional tumors are lacking [12]. In large-scale studies involving human patients, ultrasound morphological and dimensional criteria have proven reliable in differentiating adrenal lesions: tumor malignancy potential estimated on the basis of its dimensions alone showed that about 90% of malignant tumors were >40 mm in diameter [1, 14]. In the dog, only one study to date has compared the ultrasonographic appearance of adrenal lesions with their histopathological characteristics. The study was unable to establish definitive ultrasound criteria to differentiate benign from malignant lesions owing in part to the small sample size [12]. In the present study involving a large sample of dogs, we asked which ultrasound characteristics of adrenal lesions could predict for malignancy and how accurate ultrasound diagnosis (specificity and sensitivity) was as compared with histopathological diagnosis.

## Methods

For this retrospective study, we reviewed the clinical records of dogs presented to the Veterinary Teaching Hospital, University of Turin (Italy), between 2009 and 2015. Dogs for which a definitive histopathological diagnosis was available and that had undergone adrenal gland ultrasonography were included whether or not the manifestation of clinical signs was related to adrenal disease. Adrenal glands were removed and collected during adrenal glandectomy or at necropsy if the dog was euthanized. The glands were placed in 10% buffered formalin and stained with hematoxylin and eosin, Grimelius argyrophil, and Gomori trichrome stains for histopathological examination. An experienced veterinary pathologist (M.T.), unaware of the ultrasonographic findings, reviewed the macroscopic and histopathology sections of all adrenal glands.

Ultrasonography was performed using a B-mode ultrasonographic scanner (MyLab 70 X Vision machine, Esaote, Florence, Italy), with linear (7.5–12 MHz) and microconvex transducers (5.5–6.6MHz). An experienced examiner (R.Z.) carried out the ultrasonographic procedures. During abdominal ultrasonography, both adrenal glands were scanned; if lesions were detected, adrenal gland echostructure, echogenicity, dimension, laterality, number, and adjacent vascular invasion were recorded and evaluated. Lesions were classified as follows: A) homogeneous enlargement defined as normal adrenal gland shape with rounded contour; B) irregular enlargement defined as total loss of normal adrenal shape, echostructure, and dimension, with a mass aspect; C) nodular lesion defined as a round, well-defined focal parenchymal lesion, without loss of global shape; and D) multiple nodules defined as multiple, well-defined focal parenchymal lesions, without loss of adrenal global shape. Lesion size was determined by measuring the greatest dorsoventral and craniocaudal dimension in a longitudinal plane, as described by Hoerauf et al. (1999) [15]. Conventional gray-scale and color Doppler ultrasound were used to assess the adjacent vascular structures.

Statistical analysis to compare the ultrasonographic predictive parameters among benign and malignant lesions was performed by R software (version 3.1.2, R Core Team 2015). The Shapiro-Wilk normality test was used to assess normality of the data. Since data were not normally distributed, they were reported as medians and ranges. Categorical parameters (side and shape) were analysed using *χ*2 test and Fisher’s exact test, while numerical parameter (size) by Kruskal–Wallis test and Wilcoxon signed-rank test was used as post hoc test. Significance was set at *P* < 0.05. Ultrasound sensitivity and specificity in detecting adrenal lesions were calculated using 2x2 contingency tables.

Ultrasound characteristics of each lesion were compared with their corresponding histopathological characteristics. The processing concerning the description of the statistic distributions of observations was achieved by a self-developed routine implemented in the IDL 8.0 programming language (ITT Visual Information Solutions).

## Results

The clinical records of 119 dogs that had undergone ultrasound adrenal gland and histological examination were reviewed. Unilateral adrenal glandectomy was performed in 9 dogs and necropsy in 110. Histology was performed on 229 adrenal glands. Histopathological changes were noted in adrenal glands obtained from 69/119 dogs, with bilateral lesions in 36 dogs and unilateral in 33. Adrenal glands were histologically normal in 50/119 dogs.

Dogs with adrenal gland lesions, based on histopathological exam, were generally older (median age 10.5 years, range 1–17; median body weight 17.5 kg, range 2.3–52) than those with normal adrenal glands (median age 5.8 years, range 1.5–12; median body weight 23.1 kg, range 3.5–65) and equally distributed between sexes, 47.5% were male and 52.5% female. Forty-seven different breeds were included. The most commonly represented breeds were mixed breed (32), German Shepherd Dog (7), Labrador Retriever (5), Beagle (5), Boxer (5), Pit bull (4), French Bulldog (3), Bull Terrier (3), Yorkshire Terrier (3), Golden Retriever (3), Doberman Pincher (3), Schnauzer (3), Border Collie (2), Dogue de Bordeaux (2), Dachshund (2), Cocker Spaniel (2), Dalmatian (2), and Doberman (2). Other less represented breeds (31) were found. Age, sex, weight and breed were not significantly different between dogs with normal adrenal glands and pathological ones and between malignant and benign lesions.

Histological diagnosis revealed cortical hyperplasia (*n* = 67), adenocarcinoma (*n* = 17), pheochromocytoma (*n* = 10), metastases (*n* = 7), adenoma (*n* = 4), and other minor lesions (adrenalitis) (*n* = 4). The prevalence of adrenal neoplasms was 18%, of which 82.2% were primary adrenal tumors and 17.8% metastatic lesions. Ultrasound imaging to detect adrenal lesions had high specificity (100%) but low sensitivity (63.7%). Adrenal lesions were not displayed in 56.8% (62/109) of cases: 67.7% (47/62) as cortical hyperplasia, 9.6% (6/62) as cortical adenocarcinoma, 6.4% (4/62) as metastasis, 4.8% (3/62) as cortical adenoma, 1.6% (1/62) as pheochromocytoma, and 1.6% (1/62) as adrenalitis.

Table 1 presents the number, type, and characteristics of adrenal lesions identified at ultrasonography and histopathology.

**Table 1:** Ultrasonographic and histopathological features of adrenal gland lesions

Histopathological diagnosis		No. of lesions	Adrenal side			Lesion shape				Lesion size (mm)			
			L	R	Bi	A	B	C	D	<3	3.1-10	10.1-20	>20.1
Cortical Hyperplasia	US	20	6	4	10	3		17			15	5	
	AP	67	1	2	64	1	1	23	42	34	31	2	
Cortical Adenoma	US	1	1						1			1	
	AP	4	2	2				2	2		3	1	
Cortical Carcinoma	US	11	5	6		1	3	6	1			5	6
	AP	17	8	9		1	2	12	2		4	9	4
Pheochromocytoma	US	9	2	5	2		5	2	2			3	6
	AP	10	3	5	2		5	2	3		4	2	4
Metastasis	US	3		1	2			1	2	2	1		
	AP	7		1	6				7	6	1		

*US* Ultrasonographic findings, *AP* histopathological findings, *L* left side, *R* right side, *Bi* bilateral side. *A* Homogeneous enlargement, *B* Irregular enlargement, *C* Nodular lesion, *D* Multiple nodular lesions. Minor lesions (4 adrenalitis) were not express in the table because not detect by both ultrasonographic and macroscopical evaluations

**Fig. 1:**
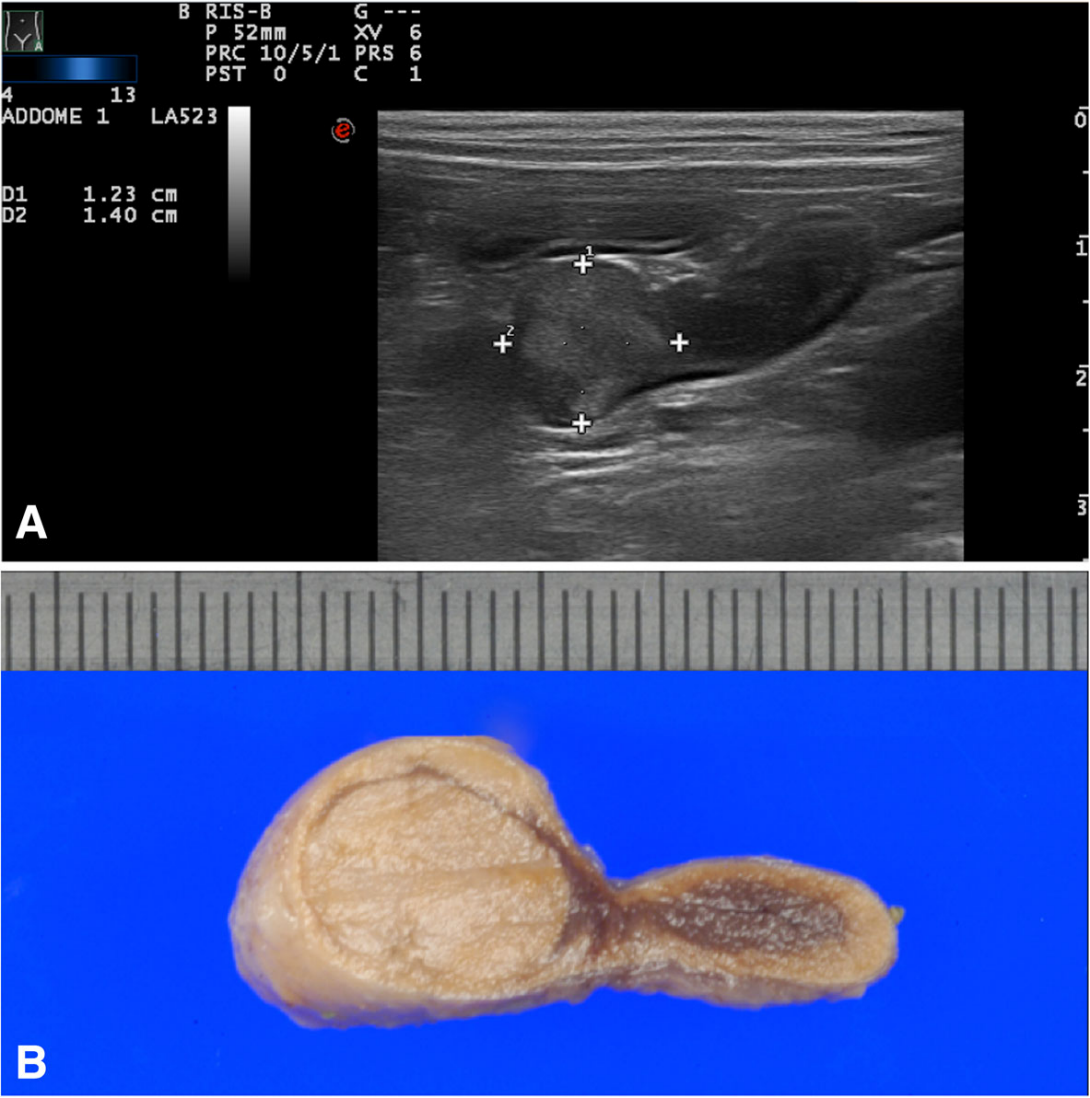
CORTICAL CARCINOMA: **a**) ultrasonographic and **b**) macroscopical images of left adrenal cortical carcinoma nodular lesion of, illustrating a left adrenal nodular lesion, 12 x14 mm size, middle uniform echogenicity, and well defined margins

Adrenal carcinoma was the most common type of adrenal primary neoplasia (17/31), and it was generally unilateral, affecting the left and right adrenal gland in 8 (47%) and 9 (53%) dogs, respectively. Adrenal glandectomy was performed in 9 dogs with histopathologically confirmed unilateral adrenal carcinoma while necroscopy was performed in the other 8 cases, in 4 of which the contralateral gland was found atrophic. The adrenal gland usually maintained its normal shape. In 82.3% (14/17) of cases the tumor presented a nodular shape (single or multiple nodules) (Fig. 1), of which 76.4% (13/17) showed a lesion >10 mm. Of note, in 2 cases ultrasound overestimated the actual lesion size. The median diameter was 15 mm (range 3-37). The echotexture was generally heterogeneous, with several small areas of inner calcification producing acoustic shadowing. In the patients with adrenocortical carcinoma, echogenicity ranged from hypo- to hyperechoic, compared to the renal cortex, and it was associated with microscopic findings of focal areas of necrosis and hemorrhage. Evidence of vascular invasion of the phrenicoabdominal vessels was noted in 23.5% (4/17) of dogs with a right-sided tumor. No invasion of the caudal vena cava or abdominal aorta was noted. Ultrasonography failed to detect lesions in 6 cases: lesions were <3 mm in 4 cases and <20 mm in 2 cases.

**Fig. 2:**
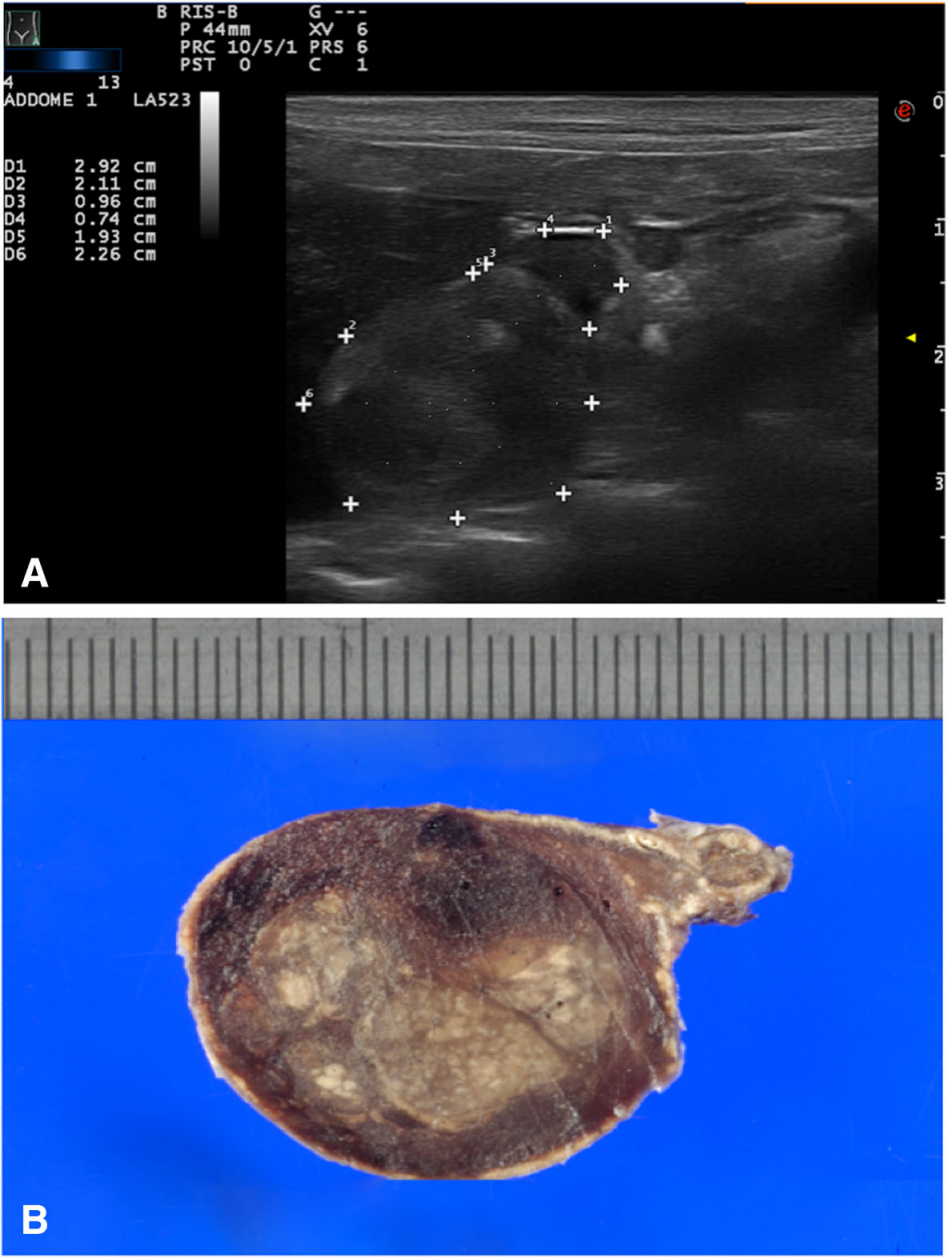
PHEOCHROMOCYTOMA: **a**) ultrasonographic and **b**) macroscopical images of right adrenal mass pheochromocytoma of, 19 x 22 mm size, heterogenic echotexture and irregular margins

Pheochromocytoma was the second most frequent primary adrenal neoplasia (10/31). In 5 cases a large, amorphous encapsulated mass, with irregular margins and loss of normal shape and parenchymal structure, was visualized (Fig. 2). The remaining lesions were solitary or multiple nodules with normal adrenal shape. In 6/10 cases, pheochromocytoma was >10 mm in diameter and in 3 cases ultrasound overestimated the actual lesion size. The median diameter was 13 mm (range 7–62). No specificity in echogenicity was noted. Tumor thrombus extending into the phrenicoabdominal vein and caudal vena cava was visualized in 40% (4/10) of cases. In 1 case, both adrenal glands were affected by a pheochromocytoma caudally displacing the kidneys, with distortion of normal renal shape. No distant metastases were found in any of these 10 dogs. Ultrasound failed to detect pheochromocytoma in only 1 case and no gross lesions were detected at necroscopy.

**Fig. 3:**
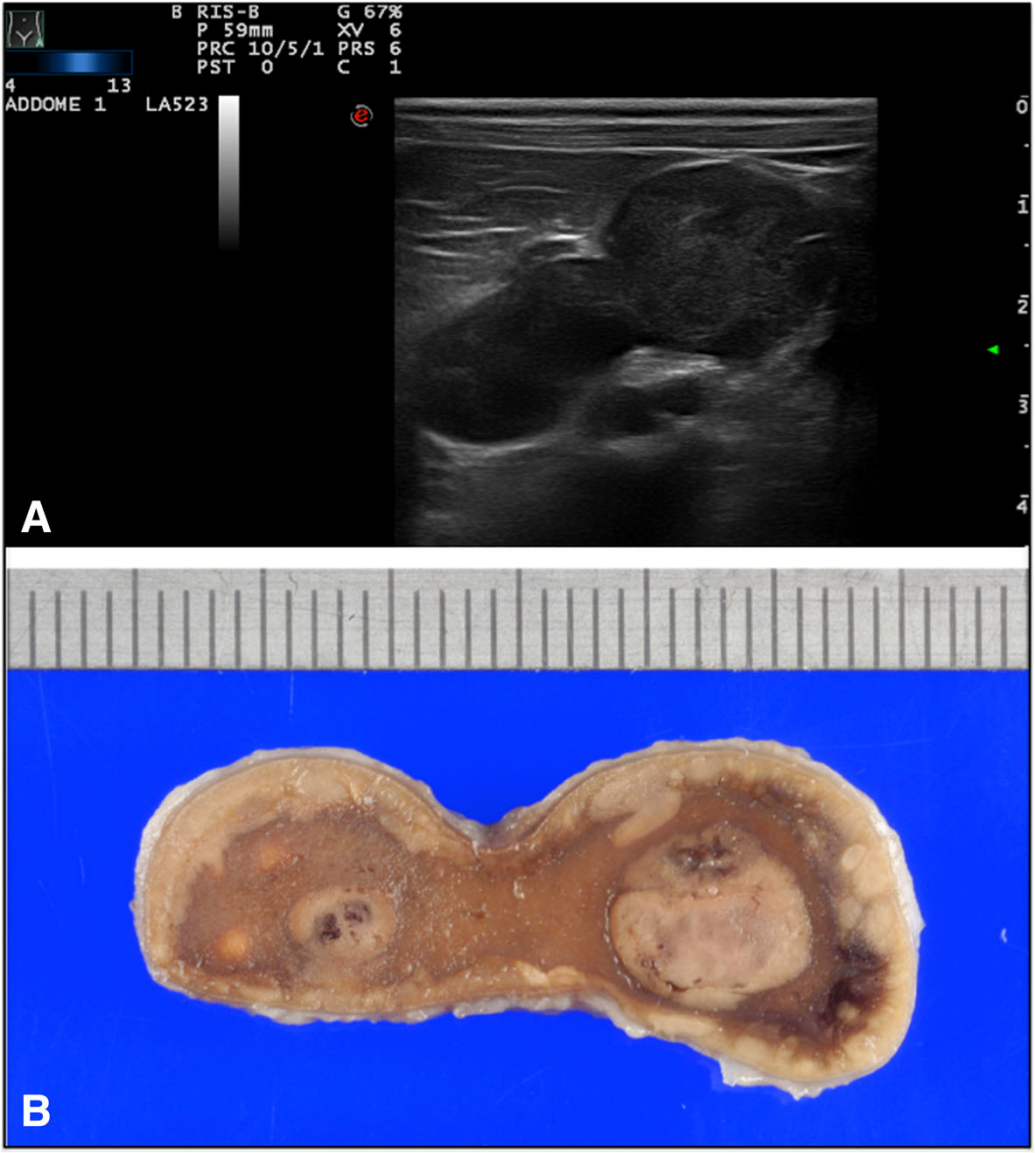
CORTICAL ADENOMA: **a**) ultrasonographic and **b**) macroscopical images of left adrenal gland cortical adenoma showing with heterogeneous multifocal nodules with small areas of necrosis, calcification, and hemorrhaging

Cortical adenoma was a rare adrenal primary neoplasia (4/31), presenting as solitary or multiple nodular lesions affecting both adrenal glands (median diameter 5.5 mm, range 4–15). The only one visualized on ultrasound appeared as heterogeneous multifocal nodules throughout the parenchymal tissue, with several small areas of inner calcification (Fig. 3).

**Fig. 4:**
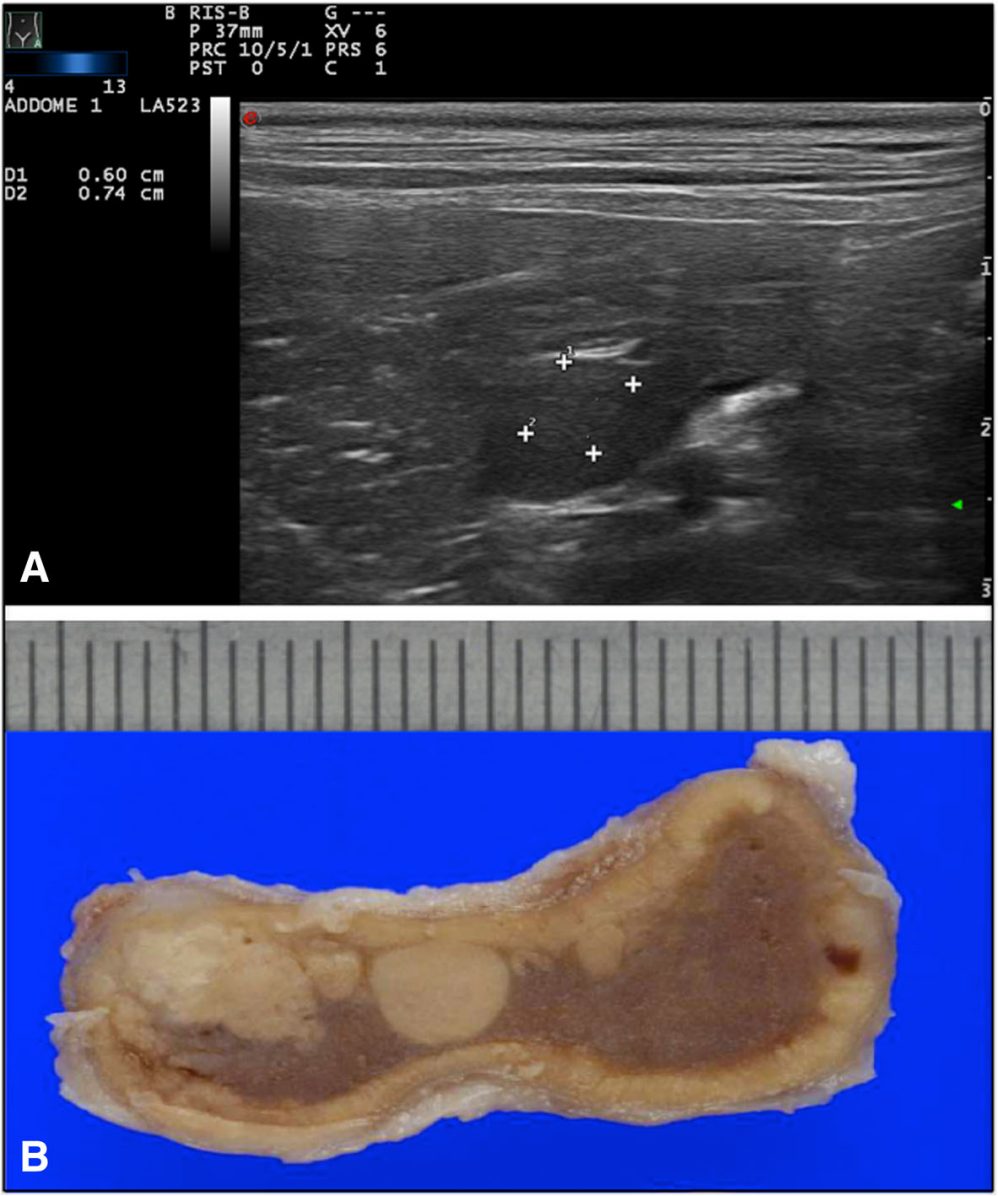
CORTICAL HYPERPLASIA: **a**) ultrasonographic and **b**) macroscopical images of left adrenal gland cortical hyperplasia with multinodular aggregated lesions

Compared with adrenocortical adenomas, the nodules in cortical hyperplasia were smaller and more numerous. Adrenocortical hyperplasia (67 cases) affected both adrenal glands in 32/35 (91%) cases. All adrenal glands affected by cortical hyperplasia maintained their normal shape. In 97% of the cases they were <10 mm and in 55% of them they had a multinodular aspect affecting the entire glandular parenchyma. Ultrasound detected cortical hyperplasia in 30% of cases; hyperplasia was characterized by focal nodules ranging from 3.1 to 10 mm in diameter (Fig. 4).

**Fig. 5:**
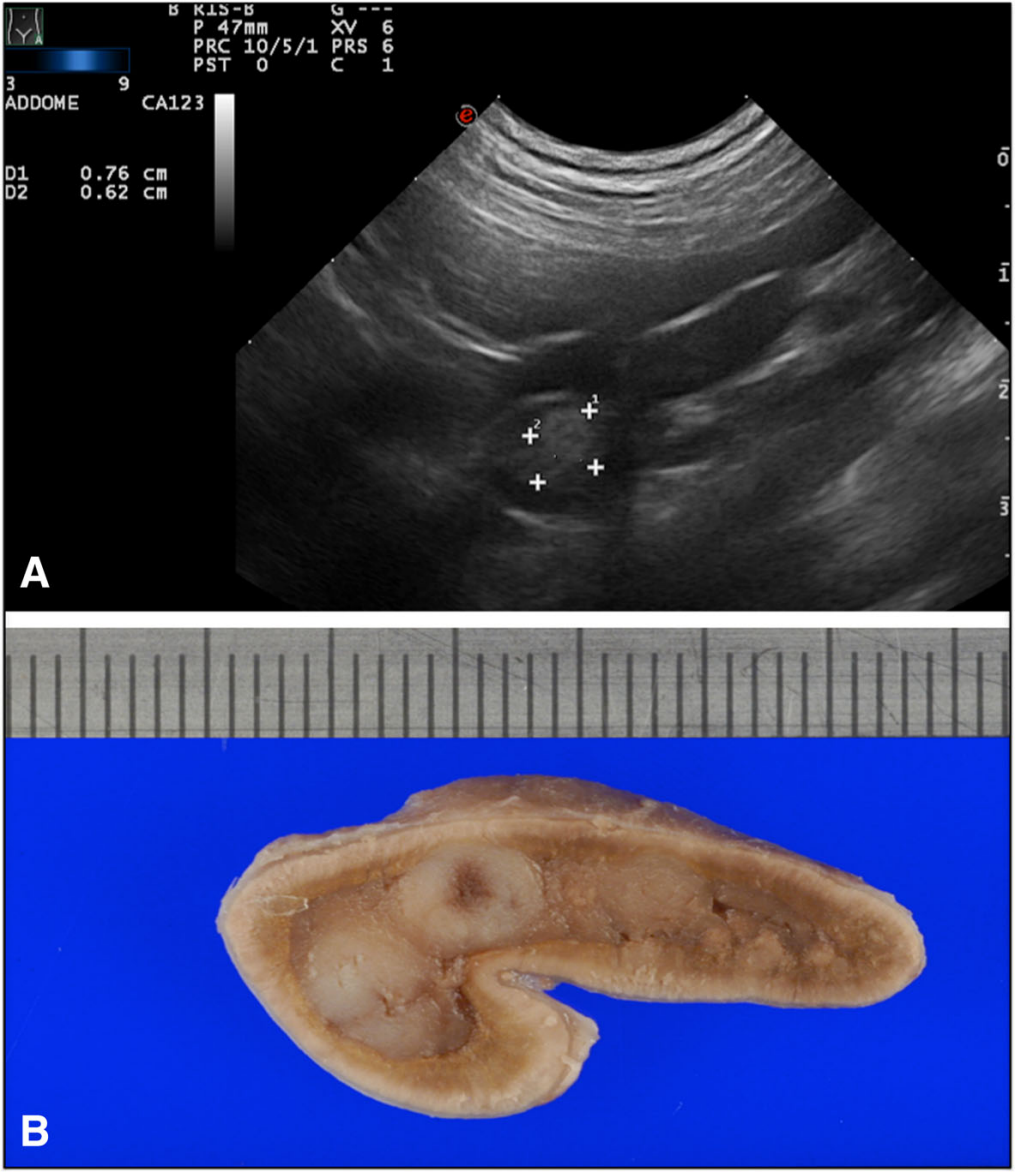
METASTASES: **a**) ultrasonographic and **b**) macroscopical images showing a right adrenal gland lung carcinoma metastases with multifocal and heterogenic nodular lesions with irregular margins

Adrenal metastasis was usually bilateral and presented as multifocal and heterogeneous nodules with irregular margins (Fig. 5). The adrenal metastases stemmed from 2 primary neoplasms: splenic hemangiosarcoma with bilateral lesions in 3 dogs and lung carcinoma in 1 dog. All metastatic lesions were <10 mm in diameter.

A comparison between the ultrasonographic predictive parameters for the diagnosis of benign (cortical hyperplasia and cortical adenoma) and malignant (cortical carcinoma, pheochromocytoma, metastasis) lesions showed significant statistical differences (*P* < 0.05) for all the parameters evaluated (lesion side, shape and size) (Table 2). Furthermore, a statistically significant association was noted between ultrasonographic nodular shape and the presence of benign lesions, such as cortical hyperplasia and cortical adenoma (*P* = 0.006), whereas an irregular enlargement of the adrenal glands was significantly associated with malignant neoplasia, such as pheochromocytoma and cortical carcinoma (*P* = 0.004). Bilaterality of adrenal lesions was significantly associated with benign lesions, such as cortical hyperplasia (*P* = 0.04) and right adrenal lesions with malignant lesions (*P* = 0.03).

**Table 2:** Ultrasonographic predictive parameters for the diagnosis of benign and malignant adrenal lesions

Histopathological diagnosis	No. of lesions	Adrenal side			Lesion shape				Lesion size
		n (%)			n (%)				Median (Range) mm
		L	R	Bi	A	B	C	D	
Benign Lesions	21	7 (50)	4 (25)	10 (71.4)	3 (75)	0 (0)	17 (65.4)	1 (16.7)	7.05 (3-16.1)
Malignant Lesions	23	7 (50)	12 (75)	4 (28.6)	1 (25)	8 (100)	9 (34.9)	5 (83.3)	10.8 (3-70.5)
p-value		1	0.03*	0.04*	0.33	0.004*	0.006*	0.18	0.0001*

*L* left side, *R* right side, *Bi* bilateral side. *A* Homogeneous enlargement, *B* Irregular enlargement, *C* Nodular lesion, *D* Multiple nodular lesions. *P <0.05

## Discussion

The prevalence of adrenal gland neoplasia in our study was 18.1%, of which 81.7% were primary adrenal neoplasia and 18.3% metastatic lesions. The most frequent neoplastic type was adrenocortical carcinoma (44.7%), followed by pheochromocytoma (26.3%), metastases (18.4%), and adrenocortical adenoma (10.5%). A high prevalence (32%) of adrenal cortical hyperplasia was also noted. As reported in humans, the majority of histologically diagnosed adrenal lesions were benign, such as cortical hyperplasia (65.1% of cases in our series) rather than neoplastic [1]. Our results are in agreement with data reported by a previous study in dogs based on histological examination showing a prevalence of 19% of adrenal neoplasia and 41.5% cortical hyperplasia [16].

Adrenal lesions are more likely to be found in older dogs [11] with a median age of 10.5 years for dogs with adrenal gland lesions and 5.8 years for those with normal adrenal glands. Although no statistically significant differences between age and type of adrenal lesions were found (*P* = 0.20), the incidence of cortical hyperplasia seemed to increase with advancing age, with 67% of the dogs showing cortical hyperplasia being ≥ 8 years old. Chronic or severe illness in old dogs may reflect an increase in adrenocortical demand in response to stress and cause adrenocortical hyperplasia. Adrenocortical hyperplasia is also described to be more frequent in small dogs, but in this study there was no significant correlation between adrenal lesion type and body weight [11].

In terms of diagnostic tools, ultrasound imaging had high specificity (100%) but low sensitivity (63.7%) in the detection of lesions. However, sensitivity seemed to improve with increasing lesion dimension. Ultrasonography failed to detect lesions <3 mm in diameter in 95% of the cases and lesions between 3 and 10 mm in 46.8% of the cases. Ultrasound failed to detect 67.7% (47/62) of cortical hyperplasia lesions and 5/7 of the metastatic lesions. In contrast, nearly all lesions (20/22) >10 mm were correctly detected.

Lesions that appear benign on ultrasound images can be confused with other types of abnormalities, including malignancy. Our data indicate that although structural features, such as lesion size, shape, laterality, and echotexture, may provide helpful diagnostic ultrasound criteria, such features alone are not necessarily pathognomonic. Of the parameters evaluated, lesion size seemed to be a distinguishing feature, as smaller adrenal lesions associated with a benign outcome whereas larger ones were more likely malignant (*P* = 0.001). Adrenal gland lesion dimension may be a useful ultrasonographic indicator to predict malignancy. Seventy percent (19/27) of adrenal primary tumors were in fact >10 mm, 30% >20 mm, and all adrenal lesions >20 mm in diameter were malignant tumors (pheochromocytoma and carcinoma both in 4 cases each). This finding is consistent with previous reports describing malignant adrenal tumors as lesions >20 mm in diameter [17–19] or 75% between 20 and 40 mm and 100% >40 mm in maximum diameter [12]. In agreement with this Cook et al. (2014) recently showed that malignancy should be strongly suspected in adrenal masses measuring >20 mm in diameter. In in such cases adrenal glandectomy seem highly recommended [11]. Similar data were reported also in human studies, where larger lesions (>40 mm) were more likely associated with malignant tumors [20]. In our study, adrenal lesions <3 mm were usually benign, with 85% (34/40) of the cases histologically diagnosed as cortical hyperplasia. However, small size can still be misleading because other small lesions may be malignant, as seen in the 6 cases of metastases. No data are available on adrenal gland lesions measuring <10 mm because this is the accepted cut-off value of adrenal gland maximum diameter set as an exclusion criterion [11].

Among the other ultrasonographic lesion features, abnormal adrenal gland shape may be a useful tool in diagnosis. Nodular shape was significantly associated with cortical hyperplasia, which was highly represented (67/119); however, all the metastatic lesions, the majority of which were cortical carcinoma (14/17), and approximately half of pheochromocytoma, showed a nodular or multinodular ultrasonographic aspects. In contrast, irregular adrenal gland enlargement was seen only in the presence of primary malignant tumors, as previously reported [12].

Adrenal laterality (right or left sides) was not found to be a useful predictor for adrenal gland abnormality, whereas bilateral lesions were significantly associated with cortical hyperplasia. As described in previous studies, however, this morphological feature could be confused with malignant metastatic lesions and with rare cases of pheochromocytoma [11, 19].

According to human and veterinary literature, vascular invasion may be a useful indicator of malignancy. Indeed, all 8 cases (4/17 cortical carcinoma and 4/10 pheochromocytoma) were diagnosed as vascular infiltration of malignant neoplastic cells, but it was not specific for the type of primary adrenal neoplasia [1, 12, 21]. In our sample, vascular invasion was a specific, but not sensitive ultrasonographic parameter. However, a possible but uncommon association between adrenal hyperplasia and vascular invasion of the distal aorta consequent to the hypercoagulability state often accompanied hyperadrenocorticism has been described [12].

Consistent with previous findings, adrenocortical carcinomas were generally unilateral, with an equal distribution between sides [22], with atrophy of the contralateral gland in 4 cases, suggesting a hormone-dependent neoplasia, as described elsewhere [2]. In agreement with previous studies [12] cortical adenomas were macroscopically characterized by a nodular pattern. In humans, they usually present as homogeneous hypoechogenic and solid focal or multifocal nodules with well-defined borders [1]. The only adenoma identified by ultrasound in our study appeared as heterogeneous multifocal nodules with several small areas of inner calcification producing acoustic shadowing. Calcification, necrosis, and hemorrhage are not microscopic features typical of adenoma but they are usually found in large lesions [12, 21]. Differentiation between cortical adenoma and adenocarcinoma based on shape, parenchymal structure, and mineralization was not possible, since 50% of adenoma and carcinoma show calcium parenchymal deposition [22].

In half of the cases of pheochromocytoma**,** the tumor was visualized as a large, amorphous encapsulated mass, with irregular margins and loss of normal shape and parenchymal structure. The remaining lesions presented as solitary or multiple nodules maintaining normal global shape. This pattern of pheochromocytoma has rarely been described [11, 12] and could be confused with other neoplastic lesions. Echogenicity was not specific for malignancy. The mixed echopatterns seemed to correlate with the microscopic evidence of hemorrhagic necrotic areas, as described by Poffenbarger et al. [18]. Larger pheochromocytoma seemed to be more heterogeneous, with predominant cystic-necrotic areas. Vascular tumor thrombus was present in 40% of cases, but without distant metastases, consistent with observations by Rosenstein [23]. In humans, pheochromocytoma is usually a benign tumor and as such is not expected to invade adjacent tissues [1]. In our study, all 10 cases of histologically diagnosed pheochromocytoma were malignant with vascular invasion, confirming its association with malignancy in dogs [12, 15].

Adrenal metastasis was frequently bilateral, presenting as multifocal and heterogeneous nodules with irregular margins. In humans, adrenal metastases vary considerably in size and infiltration pattern depending on the type of primary neoplasia from which they originate and are usually described as bilateral lesions [1]. In our series, the metastatic lesions showed similar characteristics, which could have been due to the high prevalence (85.7%) of the same primary neoplasia.

The present study has some limitations. Uncommon lesions of canine adrenal glands, such as myelolipomas, which might have led to different conclusions about ultrasound accuracy [24], were not included,. Furthermore, a high number of dogs that underwent necropsy were affected by the presence of terminal illness, which might bias the population enrolled.

## Conclusions

As the use of ultrasonography as a diagnostic screening tool continues to increase, we can expect to recognize and diagnose more cases of occult or preclinical adrenal disease. Advances in diagnostic imaging technologies will also improve the identification of small lesions (<10 mm). Although most of them were benign, such as hyperplastic nodules, they could still constitute a challenging clinical issue. Large masses or nodules (>20 mm), associated or not with vascular invasion, seemed to be a direct parameter to predict malignant adrenal gland neoplasia, while vascular invasion was a specific but not sensitive predictor of malignancy. Lesions <20 mm in diameter and with nodular shape were usually cortical hyperplastic nodules or adenomas. Other criteria, such as adrenal gland laterality, were not specific for predicting malignancy, because only the right side was significantly associated with malignant lesions and bilateral lesions, such as cortical hyperplasia, were more likely to be benign. Clinical signs and laboratory findings of hormonal hypersecretion in the differential diagnosis and workup of adrenal gland lesions require close cooperation between radiologist and endocrinologist, given that only 5–15% of adrenocortical tumors are functional and clinical signs of pheochromocytoma are often unspecific [18, 19].

## Abbreviations

A: Homogeneous enlargement
AP: Histopathological findings
B: Irregular enlargement
Bi: Bilateral side
C: Nodular lesion
D: Multiple nodular lesions
L: Left side
R: Right side
US: Ultrasonographic findings
